# Two Birds with One Stone: One-Pot Conversion of Waste Biomass into N-Doped Porous Biochar for Efficient Formaldehyde Adsorption

**DOI:** 10.3390/molecules31020201

**Published:** 2026-01-06

**Authors:** Qingsong Zhao, Ning Xiang, Miao Xue, Chunlin Shang, Yiyi Li, Mengzhao Li, Qiqing Ji, Yangce Liu, Hongyu Hao, Zheng Xu, Fei Yang, Tiezheng Wang, Qiaoyan Li, Shaohua Wu

**Affiliations:** 1 Department of Life Sciences, Changzhi University, Changzhi 046011, China; zhaoqs1983@163.com (Q.Z.);; 2 Wuhai Branch Station of Inner Mongolia Environmental Monitoring Center, Wuhai 016000, China; 3 Department of Chemistry, Changzhi University, Changzhi 046011, China; 4 College of Environment and Ecology, Taiyuan University of Technology, Jinzhong 030600, China; 5 Academy of Environmental and Resource Sciences, School of Environmental Science and Engineering, Guangdong University of Petrochemical Technology, Maoming 525000, China

**Keywords:** HCHO adsorption, N-doped biochar, DFT calculation, resource recovery

## Abstract

Converting agricultural solid waste into porous biochar for HCHO adsorption is considered as a “two birds with one stone” strategy, which can achieve the environmental goal of “treating waste with waste”. Unfortunately, the HCHO adsorption performance of pristine biochar is generally unsatisfactory, which is derived from its poor surface activity and insufficient number of pores. In this study, a series of nitrogen-doped porous biochars with adjustable N-containing groups and porosity were synthesized by one-step pyrolysis of melamine and waste jujube pit in different mass ratios (NBC-x, x represented the mass ratio of melamine to waste jujube pit, x = 4–12) for HCHO adsorption. The HCHO adsorption tests indicated that the insertion of nitrogen-containing species improved the adsorption capacity of pristine biochar (BC). However, after the insertion of excessive nitrogen-containing species, the porosity of the samples significantly decreased due to the blockage of pores, which could be disadvantageous for HCHO adsorption. DFT calculation results showed that N doping (especially pyrrolic-N) significantly increased the maxima of absolute ESP values of the carbonaceous models and consequently enhanced the affinity between polar HCHO and carbonaceous models (varied from −20.65 kJ/mol to −33.26 kJ/mol). Thus, the NBC-8 possessing both substantial nitrogen content (19.81 wt. %) and developed porosity (specific surface area of 223 m^2^/g) exhibited the highest HCHO uptake of 6.30 mg/g. This was approximately 6.4 times larger than that of BC. This work not only deepens the understanding of the HCHO adsorption mechanism at molecular scale, but also concurrently offers a facile and eco-friendly route of N-doped porous biochar preparation, an efficient technology with high-value utilization of waste biomass resources, and a sustainable method of pollution remediation.

## 1. Introduction

As a common and hazardous indoor airborne pollutant, formaldehyde (HCHO) is mainly emitted from a variety of construction/decorating materials and household products (such as particle board, flooring material, latex paint, carpet, etc.), bringing a great threat to human health [1,2]. As reported, prolonged HCHO exposure, even at the ppm level, could cause skin irritation, vomiting, asthma, emphysema, and even cancer [3,4]. Given the severe adverse impacts of HCHO on human beings, it is imperative to explore high-efficient, low-cost, and eco-friendly technologies for HCHO removal. In the past several decades, researchers have made unremitting efforts and developed multiple technologies for indoor HCHO purification, including physical adsorption [5], chemical absorption [6], biofiltration [7], plasma decomposition [8], catalytic oxidation [9], and photocatalytic oxidation [10]. Among them, physical adsorption is considered as an optimal candidate, profiting from its excellent removal efficiency, easy operation, and simple maintenance [11].

Activated carbon (AC) with highly developed porosity and abundant superficial functional groups has been widely utilized for indoor HCHO purification [12,13,14]. Nevertheless, a critical problem in the adsorption of HCHO on carbon-based adsorbents is that the physical adsorption interaction between non-polar/weak-polar carbon materials and polar HCHO is relatively weak, consequently leading to low HCHO adsorption capacity [15]. For instance, the study of Zhu et al. [16] suggested that the saturated HCHO adsorption capacity of coal-based activated carbon was merely 0.08 mg/g in spite of well-developed porosity. Analogously, Bandosz et al. [17] tested the dynamic HCHO adsorption performance of BAX (a wood-derived porous carbonaceous material), which exhibited HCHO adsorption capacities of only 0.25 mg/g and 0.23 mg/g under dry and moist conditions, respectively. Accordingly, considerable efforts have been made to introduce polar adsorption sites for improving HCHO adsorption performance.

Heteroatom doping, especially nitrogen doping, has been proven to be a simple but effective way to modulate the electronic properties of sp^2^-hybridized carbon frameworks and generate ample surface polar sites, which is expected to be advantageous for polar gas adsorption (such as HCHO) [18,19]. Unglaube et al. [20] reported that the incorporation of nitrogen atoms significantly improved the HCHO adsorption capacity of activated carbon, driven by chemical/specific interactions between the material’s newly formed N groups and HCHO. Lee et al. [21] also reported the promotional role of N doping for HCHO removal. Because of the coexistence of multiple types of N-containing groups (such as pyridinic-N, pyrrolic-N, and graphitic-N) on N-doped carbons, great difficulties arise in distinguishing the intrinsic effects of specific N-containing species for HCHO removal. This not only limits the solid understanding of HCHO adsorption mechanisms, but also hinders further optimization of N-doped porous carbon adsorbents. Hence, comprehensive understanding of the interaction between specific N-containing groups and HCHO is highly in demand.

Currently, the precursors of commercial activated carbon are mainly coals, peats, lignites, asphalts, and woods, which are nonrenewable and relatively expensive, thus increasing the operation costs of adsorption disposal [22]. Meanwhile, China is a large agricultural country which inevitably produces a large amount of agricultural solid wastes (ASWs) every year. For a long time, most ASWs have long been directly discarded or openly burned, which leads to not only serious environmental pollution problems but also to a substantial waste of biomass resources [23]. By contrast, converting ASWs into biochars for HCHO adsorption possesses dual incentives in both economic and environmental importance. In previous studies, Suresh et al. [24] prepared a pig-manure-derived biochar by ZnCl_2_ activation, which exhibited an HCHO adsorption capacity of 0.78 mg/g. Duan et al. [25] reported that bamboo-based biochar prepared by using boric acid activation achieved 80.51% HCHO removal efficiency. But to our knowledge, the application of N-doped biochar for HCHO adsorption is relatively scarce, let alone the promoting mechanism of specific nitrogen-containing groups.

In this study, a series of N-doped porous biochars (NBCs) with tunable N-containing groups and porosity were fabricated by one-pot co-pyrolysis of melamine and jujube pits at diverse mass ratios and applied to HCHO adsorption. Integrating multiple characterization methodologies and theoretical simulation analyses, we thoroughly dissected the impacts of different N-containing groups and pore structures on HCHO adsorption. This work not only deepens the comprehension of the HCHO adsorption process at a molecular level but also offers a facile and eco-friendly strategy for the rational fabrication of high-performance biochar-based adsorbents for HCHO elimination.

## 2. Results and Discussion

### 2.1. Physicochemical Properties of Biochar Adsorbents

**Figure 1:**
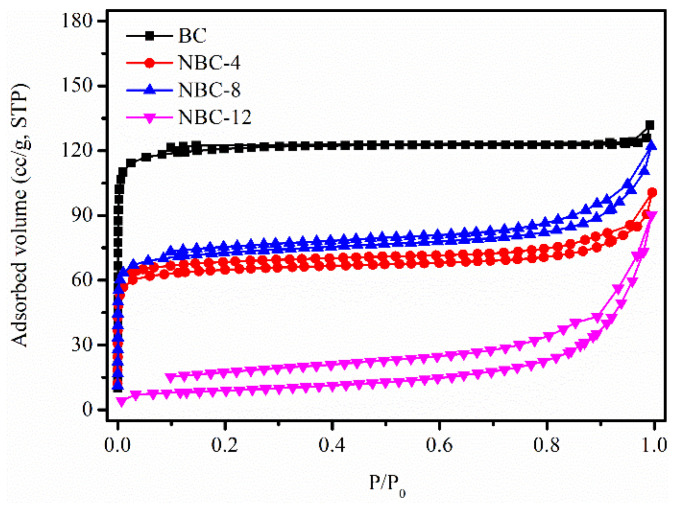
N_2_ sorption isotherms of original BC and NBC adsorbents.

To gain insight into the effect of melamine/jujube pit mass ratio on the textural features, N_2_ sorption isotherm measurements were carried out. As displayed in Figure 1, the pristine BC, NBC-4, and NBC-8 samples manifested a classical type I-like isotherm, featuring a steep nitrogen uptake at very low relative pressure (P/P_0_ < 0.05) [26]. This demonstrated that the three samples possessed developed micropores, in agreement with the pore size distribution results (Figure S1a–c). By contrast, NBC-12 presented a type IV isotherm with a H4-type hysteresis loop, which implied that the pores of this material were mainly mesopores (Figure S1d). As shown in Table 1, the S_BET_, V_tot_, and V_micro_ of the samples decreased to varying degrees after melamine modification, possibly due to pore blockage caused by the introduced N-containing groups [27]. Notably, the porosity of NBC-12 was sharply decreased, which could be unfavorable for HCHO adsorption.

The chemical compositions of biochar specimens were determined by elemental analysis (EA). As summarized in Table 1, the N content of raw BC was only 0.78 wt. %. Remarkably, the N-doping level of NBC specimens increased greatly to 13.80–23.51 wt. % when the mass ratio of melamine to jujube pits was elevated from 4 to 12. This indicated that melamine could act as a good and efficient nitrogen precursor for preparing N-doped biochar.

**Figure 2:**
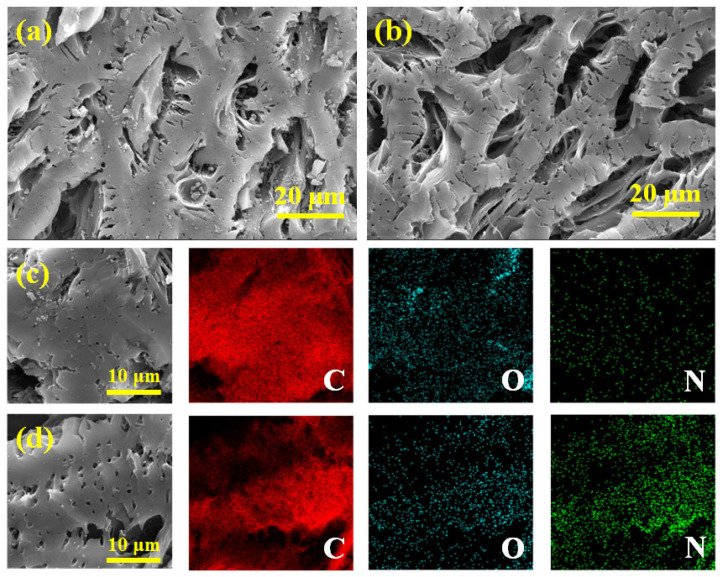
SEM micrographs and corresponding EDS mapping of BC (**a**,**c**) and NBC-8 (**b**,**d**).

FESEM was used to observe the microscopic morphologies of virgin biochar and N-doped biochar (NBC-8). The BC and NBC-8 both had a massive structure and exhibited abundant cavities and smooth surfaces (Figure 2a,b). Additionally, the EDX analysis was conducted to examine the surface elemental distribution within BC and NBC-8. Compared with the pristine BC, NBC-8 manifested an obviously denser N element signal (Figure 2c,d), further confirming the success of the N doping strategy. To further explore the microstructures of BC and NBC-8, TEM measurements were performed. As displayed in Figure S2a,b, both BC and NBC-8 were composed of thin carbon sheets and no obvious morphology/structure changes were observed after N doping. In addition, no distinct lattice fringes were detected on the BC and NBC-8, indicating poor crystallinity (Figure S2c,d).

**Figure 3:**
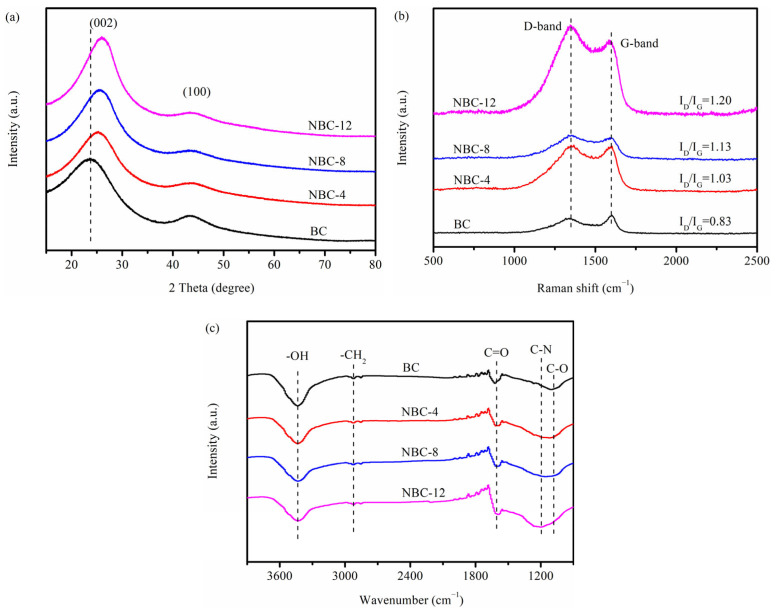
(**a**) XRD, (**b**) Raman, and (**c**) FTIR spectra of pristine BC, NBC-4, NBC-8, and NBC-12.

The crystal phase of the as-prepared BC and NBC samples was characterized via XRD and is illustrated in Figure 3a. Both the pristine biochar and N-doped biochars displayed two broad peaks at approximately 2θ = 23–26° and 43°, which were ascribed to the (002) and (100) lattice planes of graphitic-like structure carbon, respectively [19]. This suggested that the jujube pit biomass had been carbonized after pyrolysis treatment. Notably, with an increase in the melamine/jujube pit mass ratio, the (002) plane tended to shift towards a higher angle. This could be because N doping facilitated the generation of lattice defects, leading to a gradually narrowed interlayer spacing [28]. Meanwhile, the intensity of the (100) plane gradually weakened with the increase in melamine/jujube pit mass ratio, indicating that the graphite phase of biochar samples became weak after melamine modification. This was likely because a number of heterocycles/defects were incorporated into carbon matrixes after N doping, thereby destroying the ordered architecture of carbon materials.

In order to further study the local structural property of biochar adsorbents prepared with or without melamine, Raman measurements were conducted (Figure 3b). Both the pristine biochar and N-doped biochars showed two conspicuous peaks at 1349 and 1598 cm^−1^, which, respectively, corresponded to D and G bands [29]. As generally accepted, the value of I_D_/I_G_ could be utilized to characterize the degree of defects in carbon materials. With the increase in N-doping ratio, the I_D_/I_G_ ratio gradually increased from 0.83 to 1.20, implying that more structurally-defective sites were generated by incorporating N atoms into the carbonaceous matrix [30].

The FTIR spectra were collected to investigate the surface functional groups of BC and NBC samples. As depicted in Figure 3c, the peaks at approximately 2922 and 1600 cm^−1^ were derived from -CH_2_ and C=O vibrations, respectively [28,31]. The intensity of the peak at 1195 cm^−1^, originating from C-N stretching vibrations, gradually increased with the increase in melamine/jujube pit mass ratio, indicating that N atoms had been successfully incorporated into carbon matrix. Simultaneously, the intensity of peaks at 3600–3100 and 1080 cm^−1^, corresponding to -OH and C-O moieties, gradually weakened as the melamine/jujube pit mass ratio increased [32]. This could be attributed to the reaction between partial active oxygen-containing groups (e.g., -OH and C-O moieties) on the carbon materials and nitrogen-containing species (NH_3_, HCN, •NH_2_) released from the decomposition of melamine during the calcination process. While partial oxygen-containing species were consumed, some new nitrogen-containing functional groups were generated [33].

**Figure 4:**
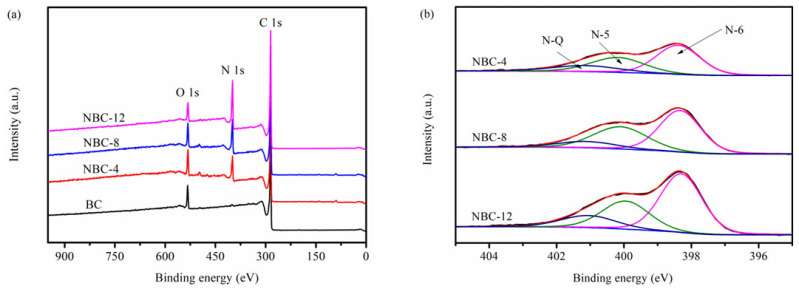
(**a**) XPS survey of samples. (**b**) N 1s scan of NBC-4, NBC-8, and NBC-12.

An XPS survey was conducted to study the effect of melamine modification on the surface composition and chemical state of biochars. As depicted in Figure 4a, the characteristic peaks of O 1s (532 eV), N 1s (399 eV), and C 1s (285 eV) were detected [34]. The surface nitrogen atomic ratios of samples (Table 2) gradually increased from 0.90 at. % to 21.30 at. % with the increase in melamine doping amount, in agreement with the trend of EA results (Table 1).

The N 1s spectra of specimens (Figure 4b and Figure S3) could be deconvoluted into three sub-peaks, corresponding to pyridinic-N (N-6, 398.4 ± 0.2 eV), pyrrolic-N (N-5, 400.0 ± 0.2 eV), and graphitic-N (N-Q, 401.0 ± 0.1 eV) [27]. As summarized in Table 2, the melamine-induced nitrogen doping led to the preferential introduction of edge-N species (i.e., pyrrolic-N and pyridinic-N). In contrast, the graphitic-N contents of N-doped biochars were lower. The main reasons could be the following: (1) nitrogen dopants prefer to react with edge species of carbon sheet and thus edge-N species are preferentially formed; (2) the annealing temperature of the biochars in this work is relatively lower (700 °C), whereas the transformation temperature of pyrrolic-N and pyridinic-N to graphitic-N is generally higher than 800 °C [35].

Figure S4 depicts the curve-fitted C 1s spectra of biochar samples. There were four sub-peaks at 284.8 ± 0.1, 285.8 ± 0.2, 286.5 ± 0.1, and 289.0 ± 0.2 eV, which belonged to C-C, C-N, C-O, and C=O, respectively [28]. Obviously, the relative ratio of C-N species gradually increased with the increase in the melamine/jujube pit mass ratio. The above results further confirmed the successful introduction of N elements.

### 2.2. Dynamic HCHO Adsorption Ability Evaluation

**Figure 5:**
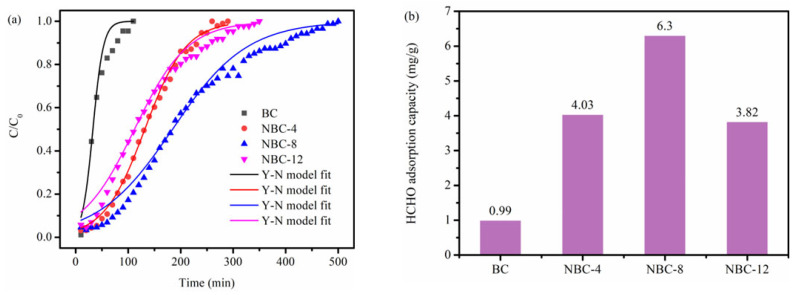
(**a**) Breakthrough profiles and (**b**) corresponding saturated uptakes of original BC and NBC adsorbents for HCHO adsorption.

The dynamic HCHO adsorption capability of biochar samples was assessed by breakthrough curves. As displayed in Figure 5a,b, the pristine BC sample demonstrated inferior HCHO adsorption ability, corresponding to a saturated uptake of only 0.99 mg/g. By contrast, the saturated uptake of these NBC samples was remarkably improved. Of note, the saturated uptake of NBC samples first increased then decreased with the increase in melamine/jujube pit mass ratio, suggesting that moderate melamine doping could effectively enhance the HCHO adsorption ability of biochar adsorbents. Moreover, NBC-8 possessed the superior HCHO removal ability, corresponding to an adsorption saturation time of 500 min, with an enormous saturated uptake of 6.30 mg/g. Furthermore, the NBC-8 synthesized in this work was benchmarked against various HCHO adsorbents reported in the literature (Table S1). The comparison results revealed that its saturated HCHO uptake capacity also outperformed that of previously reported adsorbents tested under analogous experimental conditions.

To estimate the HCHO adsorption kinetics of biochars, the breakthrough profiles were fitted using the Yoon–Nelson (Y-N) model [36], which described the adsorption process as follows:

$$t=T_{50}+\frac{1}{k}\ln\frac{C_{t}}{C_{0}-C_{t}}$$

where T_50_ is the required time at which the outlet HCHO concentration reaches half of the inlet HCHO concentration, k stands for the rate constant, and C_0_ and C_t_ are, respectively, the inlet HCHO concentration and effluent HCHO concentration at time t (min).

As displayed in Figure 5a and Table S2, the dynamic HCHO adsorption curves of specimens could be well fitted by the Y-N model (R^2^ > 0.935). These N-modified samples had a lower rate constant k compared with the pristine counterparts. This could be because of the partial pore blockage by the introduced nitrogen-containing functional groups, which hindered the diffusion of HCHO.

**Figure 6:**
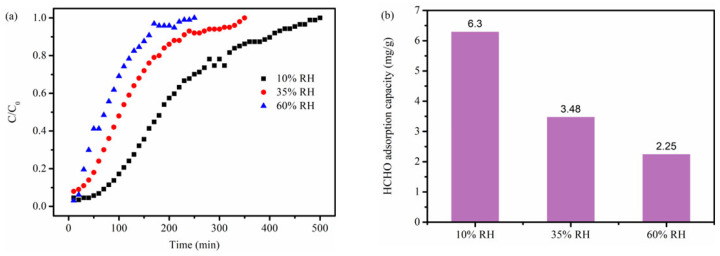
(**a**) Breakthrough profiles and (**b**) corresponding saturated uptakes of NBC-8 adsorbent at different RH.

It is necessary to assess how relative humidity (RH) impacts the formaldehyde adsorption performance of NBC-8 given that there exists a certain amount of water vapor in the real indoor environment. The saturated uptakes of NBC-8 declined progressively with increasing RH (Figure 6). The phenomenon may stem from moisture competing with HCHO for adsorption sites [20,37]. Nevertheless, NBC-8 retained a 2.25 mg g^−1^ formaldehyde uptake at higher relative humidity (60% RH), which was still significantly superior to that of pristine BC with 0.99 mg g^−1^ formaldehyde uptake at 10% RH.

**Figure 7:**
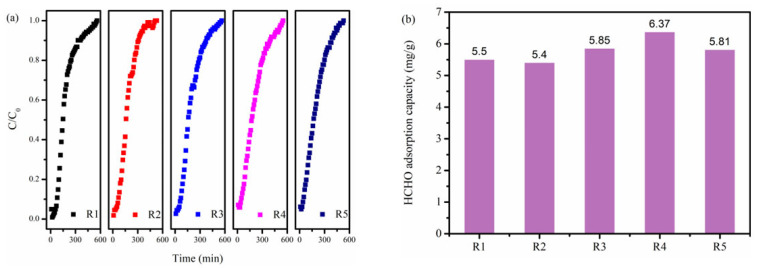
(**a**) Breakthrough profiles and (**b**) corresponding saturated uptakes of regenerated NBC-8 adsorbent.

The regeneration performance of adsorbent is also critical from the view of economics and sustainable development. As displayed in Figure 7, the regenerated NBC-8 showed no significant decline in breakthrough time and adsorption capability, and the 5th regenerated NBC-8 still retained ca. 92% of its initial HCHO adsorption capacity. In addition, the morphology of the 5th regenerated NBC-8 was similar to that of the fresh NBC-8 sample and no obvious morphology change was observed after thermal regeneration (Figure S5). This indicates that the as-synthesized NBC-8 adsorbent possesses excellent recyclability and has potential for practical application.

### 2.3. Structure–Performance Relationship Analysis

To fully gain insight into the influence of biochar physicochemical properties on HCHO adsorption capacity, the correlations of porosity (S_BET_, V_tot_, and V_micro_) as well as N content with saturated adsorption capacity were established. As displayed in Figure S6a–d, the relationships between HCHO adsorption capacity and S_BET_, V_tot_, V_micro_, or N content are rather scattered and irregular, which implies that the HCHO adsorption capacity of these biochars could not be determined by any single factor. For example, although the pristine BC possesses the maximum S_BET_, V_tot_, and V_micro_, its HCHO adsorption capacity is the lowest among them (Figure 5b and Table 1). This could be due to the fact that its N content is the lowest, which is unfavorable for the specific adsorption of polar HCHO on the biochars. On the other hand, although NBC-12 possesses the highest nitrogen content, its HCHO capacity is inferior to those of NBC-4 and NBC-8, with middling N content. This may be because the porosity of NBC-12 is the poorest (Table 1) and thus the HCHO adsorption over NBC-12 is significantly inhibited. Notably, the NBC-8 with the second highest porosity and nitrogen content exhibited the optimal HCHO adsorption performance. Another sample with relatively high HCHO adsorption capacity (i.e., NBC-4) also possessed both medium N content and porosity. Thus, it can be concluded that the HCHO adsorption performance of the biochars prepared in this work was determined by the synergistic effect of N content and porosity.

### 2.4. Theoretical Calculation

The above results suggest that nitrogen doping can promote HCHO adsorption, but the specific mechanism is still unclear. To study the effect of different N-containing species on HCHO adsorption, we fabricated four carbon sheet models, including undoped carbon sheet and pyrrolic-N-, pyridinic-N-, and graphitic-N-decorated carbon sheets (Figure S7), and studied HCHO adsorption behavior on these models by DFT calculation.

**Figure 8:**
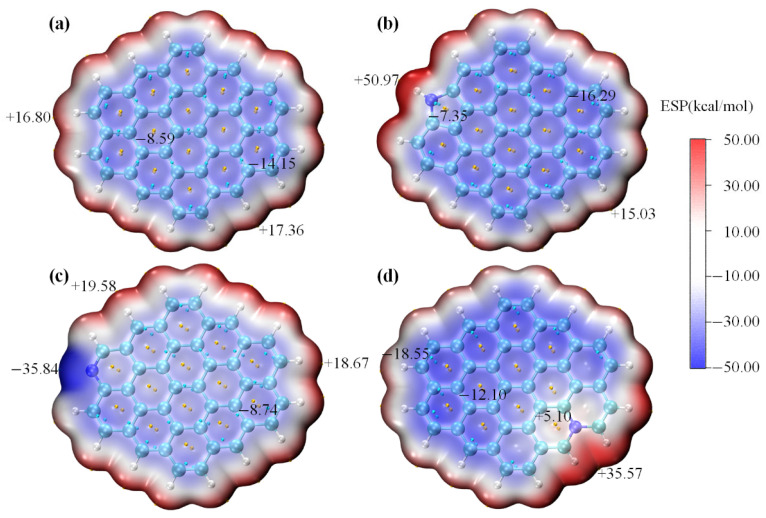
ESP analysis of perfect graphite (**a**), N-5-modified graphite (**b**), N-6-modified graphite (**c**) and N-Q-modified graphite (**d**).

Electrostatic potential (ESP) maps of the undoped carbon sheet and N-decorated carbon sheets are shown in Figure 8a–d. The ESP distribution of perfect graphite was comparatively uniform (−14.15 to 17.36 kcal/mol), suggesting its weak polarity. This implied that the intermolecular interaction between perfect graphite and polar HCHO was expected to be relatively weak. Of note, the introduction of N atoms remodeled the charge distribution of the carbon sheet model and significantly increased the inhomogeneity of ESP distribution. For the pyrrolic-N-, pyridinic-N-, and graphitic-N- doped carbon sheets, their ESP distributions ranged from −16.29 to 50.97 kcal/mol, −35.84 to 19.58 kcal/mol, and −18.55 to 35.57 kcal/mol, respectively. The below ESP analysis clearly demonstrated that N-modifying (notably pyrrolic-N) increased the polarity of the carbon sheet model, which should be conducive to polar HCHO adsorption.

**Figure 9:**
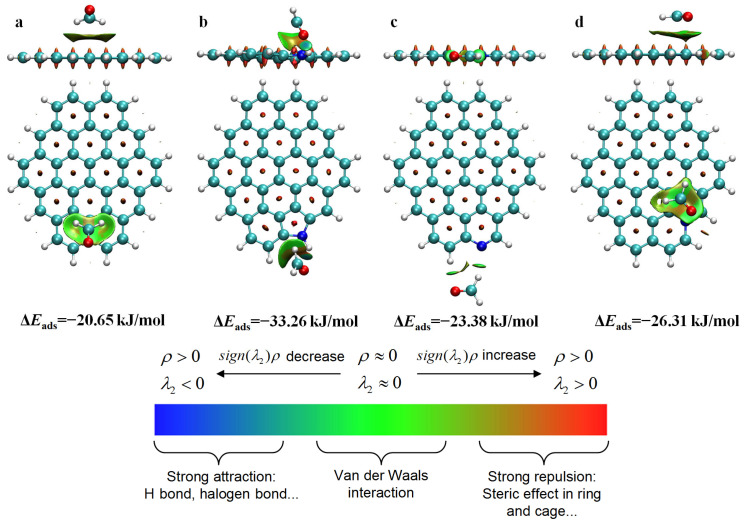
Side and top views of RDG analysis of HCHO adsorption over perfect graphite (**a**), N-5-modified graphite (**b**), N-6-modified graphite (**c**), and N-Q-modified graphite (**d**).

Reduced Density Gradient (RDG) analysis could be used to understand noncovalent interactions between adsorbate and adsorbent [38]. As shown in Figure 9a–d, green isosurfaces were formed between HCHO and the undoped carbon sheet and N-decorated carbon sheets, indicating that their interactions fell between van der Waals interactions and hydrogen bonding. It was obvious that the HCHO adsorption energy on the carbon sheets modified with N-containing functional groups was significantly higher than that of perfect graphite (−20.65 kJ/mol). For the undoped carbon sheet model, the electron-deficient C and H atoms of HCHO tended to interact the plane of the benzene ring with the π electron cloud, and the corresponding HCHO adsorption energy was −20.65 kJ/mol. For the N-5-decorated carbon sheet model, the electron-rich O atom of HCHO interacted with the electron-deficient H atom attached to the pyrrolic-N group, and the corresponding HCHO adsorption energy was significantly enhanced to −33.26 kJ/mol. For the N-6-decorated carbon sheet model, the electron-deficient H atom of HCHO was liable to interact with the electron-rich pyridinic-N atom, corresponding to the HCHO adsorption energy of −23.38 kJ/mol. For the N-Q-decorated carbon sheet model, the HCHO tended to parallelly adsorb over the benzene ring connected with the N-Q atom, corresponding to the HCHO adsorption energy of −26.31 kJ/mol. Among all configurations, the pyrrolic-N-decorated carbon model possessed the strongest adsorption affinity for HCHO (−33.26 kJ/mol), possibly because of its significantly stronger polarity (Figure 8b). This explained why the HCHO adsorption performance was enhanced after N doping and also verified the experimental results.

Figure 9 and Figure S8 reveal that HCHO and H_2_O had analogous adsorption sites on pristine and N-doped carbon models and their adsorption energies were close, verifying their competitive adsorption behavior.

## 3. Experimental Section

### 3.1. Materials

Jujube pit was sourced from Changzhi City, Shanxi Province, China, which was crushed into 0.45–0.90 mm particles for later use. Melamine (AR, ≥99.5%) was purchased from Damao Chemical Reagent Factory (Tianjin, China).

### 3.2. Preparation of Biochars

Firstly, the 3 g of jujube pits and melamine at different mass ratios (melamine/jujube pit mass ratios = 0, 4, 8, and 12, respectively) were mixed and ground in a mortar for 0.5 h. Subsequently, the blend was annealed at 700 °C for 2 h at a heating rate of 5 °C/min under an argon atmosphere. The obtained samples were washed three times thoroughly with ultrapure water and then dried at 105 °C for 12 h. The as-synthesized materials were, respectively, named BC, NBC-4, NBC-8, and NBC-12, according to their melamine/jujube pit mass ratios. The total yield of these biochars is in the range of 29.74–55.57% (Table S3).

### 3.3. Characterization

The details about characterization are listed in the Supplementary Materials.

### 3.4. HCHO Adsorption Tests

Typically, 300 mg of biochar (with a particle size of 0.30–0.45 mm) was packed in a quartz reactor (i.d. = 6 mm), which was equipped with a water jacket to maintain a constant temperature at 30 ± 0.1 °C. All gas flows were controlled by mass flow controllers. The composition of reactant gas was 10 ppm HCHO, 10% relative humidity (RH), and 20% O_2_ and N_2_ balance. The total flow rate was 780 mL min^−1^, corresponding to a weight hourly space velocity (WHSV) of 156,000 mL g^−1^ h^−1^. The inlet and outlet HCHO concentrations were analyzed using a FTIR gas analyzer (Gasmet Instruments DX-4020, Vantaa, Finland). The HCHO adsorption capacities were calculated according to the following equations:

$$q_{t}=\frac{Q\cdot M_{\mathrm{HCHO}}\cdot \int_{0}^{t_{s}}(C_{0}-C_{t})\mathrm{dt}}{1000{\cdot R}_{g}\cdot m}$$

where q_t_ represents the HCHO adsorption capacity (mg HCHO/g carbon), Q is the flow rate of feed gas (0.78 L/min), M_HCHO_ is the molar mass of HCHO (30 g/mol), t_s_ represents the reaction time when reaching the breakthrough point, C_0_ is the inlet HCHO concentration (ppm), C_t_ is the outlet HCHO concentration (ppm) at t moment, R_g_ is the gas molar volume at 30 °C and 101 kPa (24.9 L/mol), and m is the adsorbent mass (0.3 g).

To assess the influence of RH on the HCHO uptake of NBC-8, the RH of feed gas was adjusted to 35% and 60%, and other reaction conditions were identical to those of the aforementioned HCHO adsorption experiments.

The reusability of NBC-8 was measured by testing NBC-8 after 400 °C thermal regeneration in a N_2_ atmosphere for 60 min, and the reaction conditions were identical to those of the aforementioned HCHO adsorption experiments.

### 3.5. Theoretical Calculation Details

The theoretical calculation details are displayed in the Supplementary Materials.

## 4. Conclusions

Herein, we successfully fabricated a series of N-doped biochars with adjustable N-containing groups and porosity by one-pot co-pyrolysis of melamine and jujube pits at diverse mass ratios. The experiments and DFT calculations were combined to study the relationship between N-containing groups, pore structure, and HCHO adsorption performance. The experimental results displayed that N doping significantly enhanced the HCHO uptake of biochar. However, after the insertion of excessive nitrogen-containing species, the porosity of samples significantly decreased due to the blockage of pores, which could be disadvantageous for HCHO adsorption. The calculation results suggested that the introduction of nitrogen moieties (especially pyrrolic-N) enhanced the polarity of the carbon sheet and consequently strengthened the affinity between polar HCHO and carbonaceous models. Finally, benefiting from the joint effects of the high N content and developed porosity, NBC-8 exhibited the optimal HCHO adsorption ability. This work not only deepens the understanding of the HCHO adsorption mechanism but also provides a facile and eco-friendly strategy for fabricating high-performance N-doped biochar for pollution remediation applications, thus realizing the environmental goal of “treating waste with waste”.

## Figures and Tables

**Table 1:** Pore structural parameters and nitrogen contents of original BC and NBC adsorbents.

Sample	S_BET_ (m^2^/g)	V_tot_ (cm^3^/g)	V_micro_ (cm^3^/g)	N Content (wt. %)
BC	364	0.201	0.168	0.78
NBC-4	197	0.146	0.096	13.80
NBC-8	223	0.183	0.106	19.81
NBC-12	31	0.131	0.001	23.51

**Table 2:** Surface atomic composition of biochars quantified via XPS.

Sample	C (at. %)	O (at. %)	N (at. %)	N-5 (at. %)	N-6 (at. %)	N-Q (at. %)
BC	92.13	6.97	0.90	0.22	0.38	0.30
NBC-4	80.07	7.73	12.19	3.92	6.25	2.02
NBC-8	77.35	6.81	15.84	5.77	8.25	1.82
NBC-12	73.26	5.44	21.30	6.36	11.72	3.22

## Data Availability

Data will be made available on request.

## Supplementary Materials

The following supporting information can be downloaded at https://www.mdpi.com/article/10.3390/molecules31020201/s1. Figure S1. Pore size distribution curves of (a) BC, (b) NBC-4, (c) NBC-8 and (d) NBC-12. Figure S2. TEM and HRTEM micrographs of BC (a, c) and NBC-8 (b, d). Figure S3. High-resolution N 1s spectra of pristine BC. Figure S4. High-resolution C 1s spectra of pristine BC, NBC-4, NBC-8 and NBC-12. Figure S5. SEM (a) and TEM (b, c) micrographs of 5th regenerated NBC-8. Figure S6. HCHO adsorption capacity of samples versus their (a) S_BET_, (b)V_tot_, (c) V_micro_ and (d) nitrogen content. Figure S7. Carbon sheet models (a) without N atom, (b) with a pyrrolic-N, (c) with a pyridinic-N, and (d) with a graphitic-N. Figure S8. Side and top views of RDG analysis of H_2_O adsorption over perfect graphite (a), N-5 modified graphite (b), N-6 modified graphite (c) and N-Q modified graphite (d). Table S1. HCHO adsorption capacity comparison of NBC-8 and some reported adsorbents. Table S2. The Yoon-Nelson model fitting parameters of pristine BC and NBCs for HCHO adsorption. Table S3. The total yield of pristine BC and NBCs. References [5,16,17,24,38,39,40,41,42,43,44] are cited in the Supplementary Materials.
