# Supplementary material for: Two Birds with One Stone: One-Pot Conversion of Waste Biomass into N-Doped Porous Biochar for Efficient Formaldehyde Adsorption
Source: Molecules. 2026 Jan 6;31(2):201. doi: 10.3390/molecules31020201 (PMC12844407; doi:10.3390/molecules31020201)
Supplement: Supplementary file 1 [file molecules-31-00201-s001.zip › molecules-3967373-supplementary.pdf]

Supplementary Materials for

Two Birds with One Stone: One-Pot Conversion of Waste Biomass into N-Doped Porous Biochar for Efficient Formaldehyde Adsorption

Qingsong Zhao[1], Ning Xiang[1]*, Miao Xue[2], Chunlin Shang[2], Yiyi Li[1], Mengzhao Li[3], Qiqing Ji[1], Yangce Liu[1], Hongyu Hao[1], Zheng Xu[1], Fei Yang[1], Tiezheng Wang[1], Qiaoyan Li[4]*, Shaohua Wu[5]*

[1] Department of Life Sciences, Changzhi University, Changzhi 046011, China

[2] Wuhai Branch Station of Inner Mongolia Environmental Monitoring Center, Wuhai 016000, China

[3] Department of Chemistry, Changzhi University, Changzhi 046011, China

[4] College of Environment and Ecology, Taiyuan University of Technology, Jinzhong 030600, China

[5] Academy of Environmental and Resource Sciences, School of Environmental Science and Engineering, Guangdong University of Petrochemical Technology, Maoming, 525000, China.

*Corresponding author.

E-mail: x18270911586@163.com, liqiaoyan@tyut.edu.cn, wushaohua@hnu.edu.cn.

Characterization

N_2 adsorption-desorption isotherms were determined using a Beishide 3H-2000PS2 sorption analyzer (Beishide, Beijing, China) at 77 K. The chemical composition was determined using an UNICUBE elemental analyzer (Elementar, Frankfurt, Germany). The morphologies and element distribution of samples were observed by the Apreo 2 FESEM (Thermo Scientific, Waltham, MA, USA) equipped with the X-max energy dispersive spectrometer (Oxford instruments, Oxford, Britain). TEM and HRTEM measurements were conducted on the JEM-2100F (JEOL, Tokyo, Japan). PXRD measurements were conducted on a Rigaku Smartlab9 X-ray diffractometer (Rigaku, Tokyo, Japan) with Cu Kα radiation (λ = 1.5418 Å) operated at 40 kV and 40 mA. The Raman spectra were recorded on a HORIBA LabRAM HR Evolution Raman spectrometer (Horiba, Paris, France) using a 532 nm He-Ne laser. The FTIR spectra were collected on a Nicolet iS10 FTIR spectrometer (Thermo Scientific, Waltham, MA, USA). XPS spectra of samples were recorded on an ESCALAB 250Xi photoelectron spectroscopy (Thermo Scientific, Waltham, MA, USA) with an Al Kα X-ray source (1486.6 eV). The binding energy (B.E.) of spectra was calibrated by the C 1s peak (284.8 eV).

Theoretical calculation details

The aromatic ring clusters are often adopted in the DFT calculation of carbon materials. Studies have shown that the predicted molecular properties were independent of the number of aromatic rings when the aromatic ring cluster consisted of no less than six aromatic rings [38, 39]. Based on a reasonable compromise between computational expense and accuracy, the carbonaceous models with and without N atoms were established based on seven fused rings to calculate the influence of N-containing functional groups on HCHO/H_2O adsorption and the models are shown in Figure S7.

Gaussian 16 was employed to complete all calculations [40]. Density functional theory (DFT) is used to calculate the binding mode between the basic unit of materials and HCHO/H_2O. Geometric optimization and single-point energy calculations were performed at the B3LYP and M062X levels, respectively, using 6-31G* calculations, and dispersion correction with D3 (BJ). The adsorption energy of HCHO/H_2O on the surface of carbon sheet is calculated by the following formula:

$$\Delta E_{ads} = E_{complex} - (E_{HCHO} + E_{carbon}) \qquad (S1)$$

$$\Delta E'_{ads} = E'_{complex} - (E_{H2O} + E_{carbon}) \qquad (S2)$$

Where E_{HCHO}, E_{carbon}, E_{H2O}, $E_{complex}$ and $E'_{complex}$ are the energy of HCHO single molecule, carbon sheet single molecule, H_2O single molecule, carbon sheet molecule associated with HCHO and carbon sheet molecule associated with H_2O, respectively.

The electrostatic potential method was used to investigate the most possible adsorption sites. Noncovalent interaction analysis (NCI) was employed to analyze noncovalent interactions. The reduced density gradient (RDG) isosurfaces (s=0.5 a.u.) were colored on a blue–green–red scale according to values of sign(λ_2)ρ, ranging from –0.05 to 0.05 a.u. A blue isosurface implied a strong attractive interaction, while green represented van der Waals interactions and red indicated a strong nonbonding interaction. RDG calculations were performed by Multiwfn [41]. The visualization of weak interactions in this study was performed through the VMD software [42].

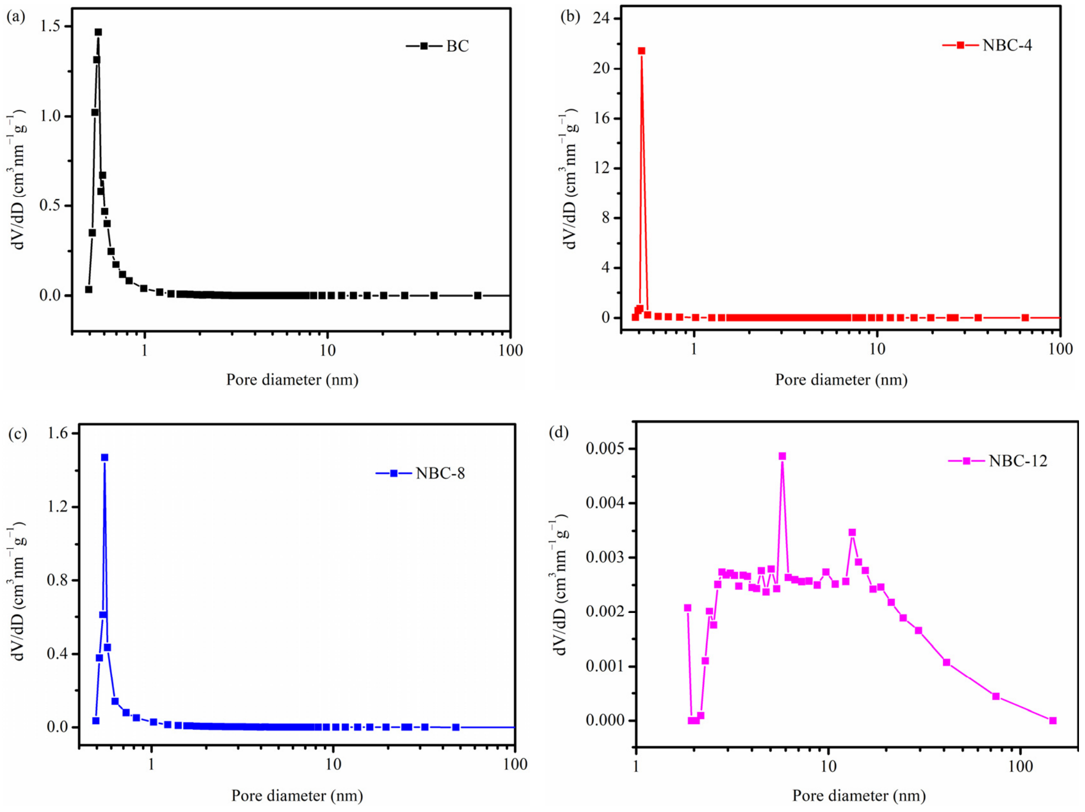

Figure S1. Pore size distribution curves of (a) BC, (b) NBC-4, (c) NBC-8 and (d) NBC-12

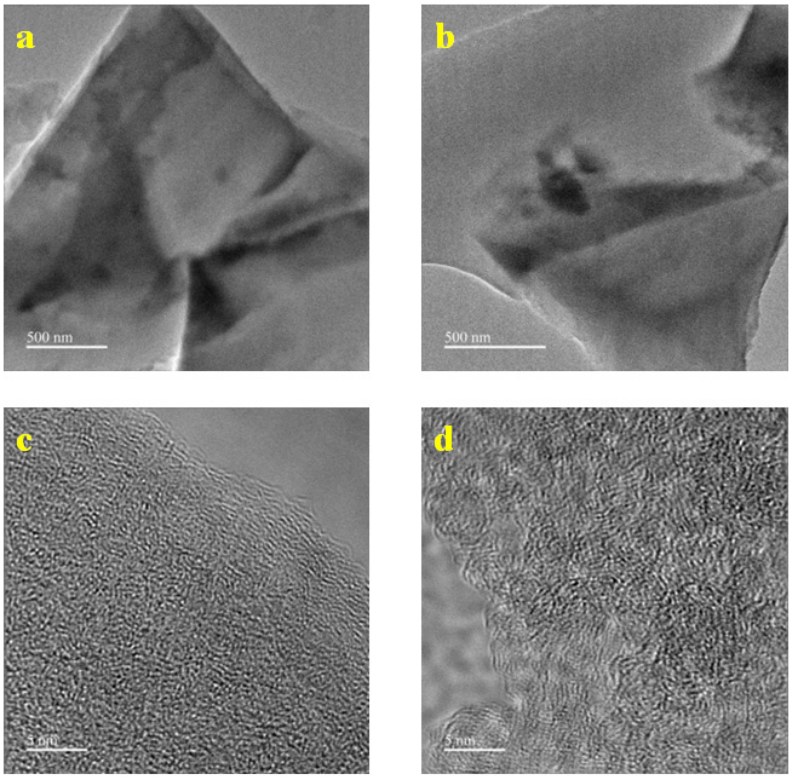

Figure S2. TEM and HRTEM micrographs of BC (a, c) and NBC-8 (b, d).

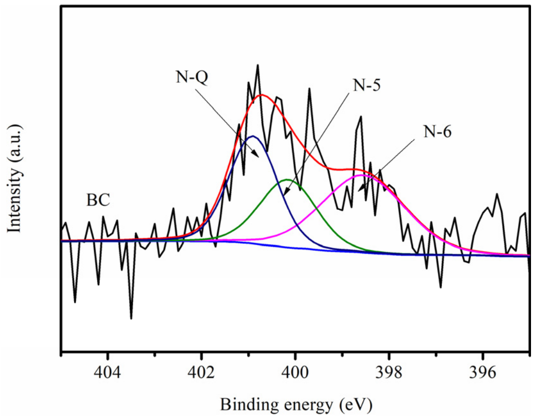

Figure S3. High-resolution N 1s spectra of pristine BC.

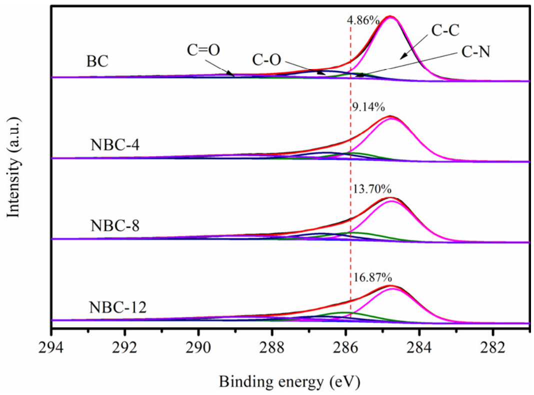

Figure S4. High-resolution C 1s spectra of pristine BC, NBC-4, NBC-8 and NBC-12.

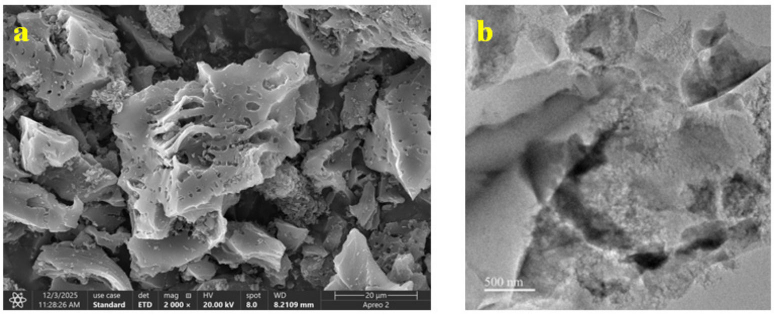

Figure S5. SEM (a) and TEM (b, c) micrographs of 5th regenerated NBC-8.

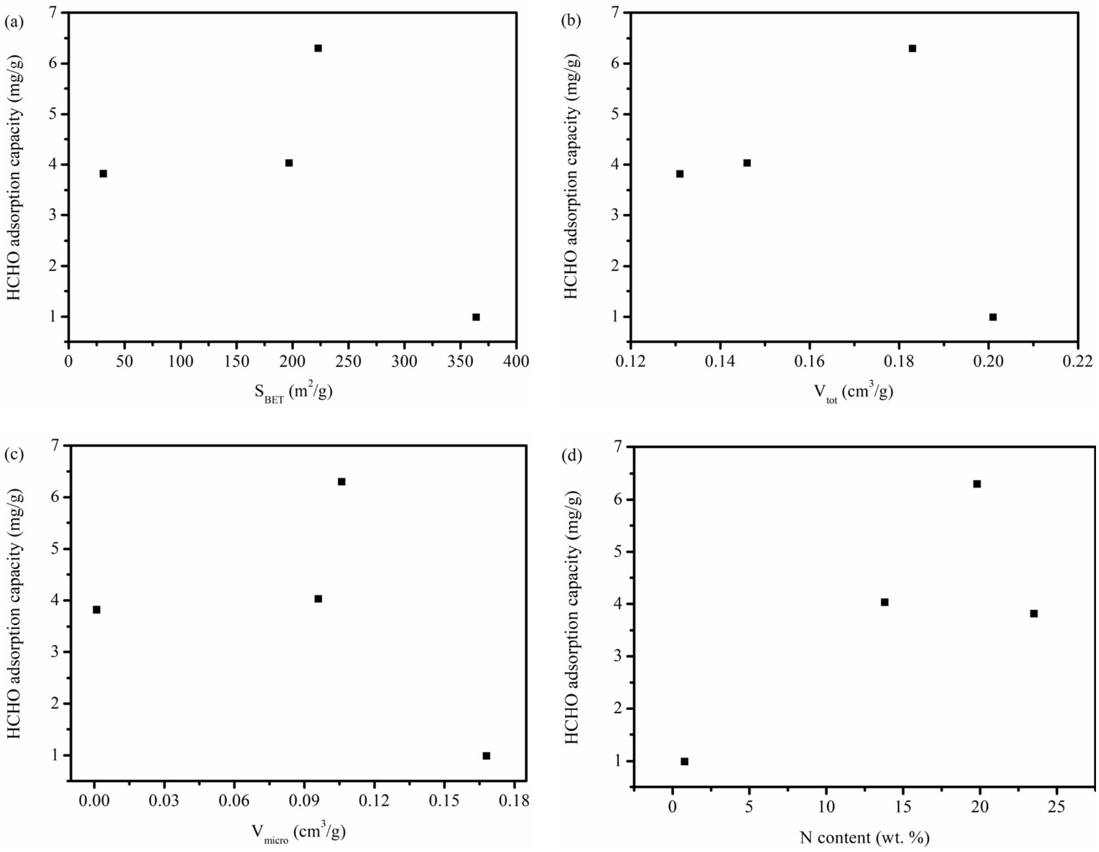

Figure S6. HCHO adsorption capacity of samples versus their (a) S_{BET}, (b)V_{tot}, (c) V_{micro} and (d) nitrogen content.

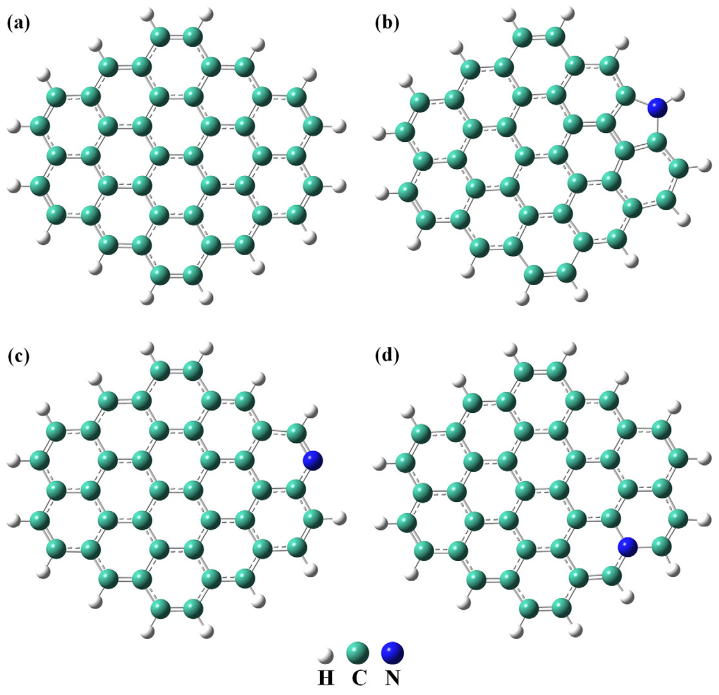

Figure S7. Carbon sheet models (a) without N atom, (b) with a pyrrolic-N, (c) with a pyridinic-N, and (d) with a graphitic-N.

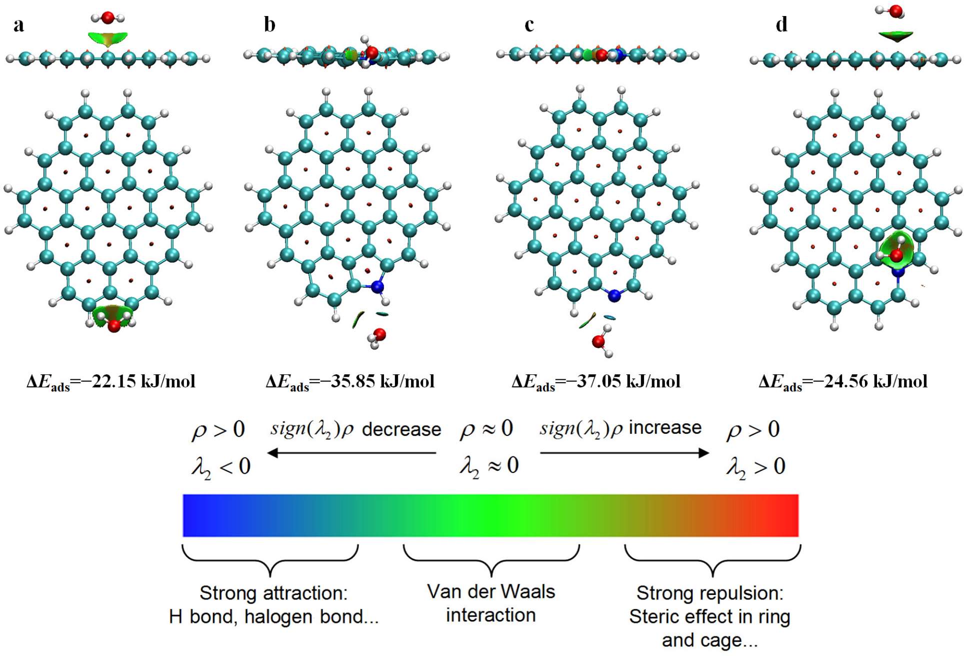

Figure S8. Side and top views of RDG analysis of H_2O adsorption over perfect graphite (a), N-5 modified graphite (b), N-6 modified graphite (c) and N-Q modified graphite (d).

Table S1. HCHO adsorption capacity comparison of NBC-8 and some reported adsorbents.

Adsorbent	Adsorbent precursors	Test conditions	HCHO adsorption capacity (mg HCHO/g adsorbent)	Ref.
NBC-8	jujube pit	~10 ppm HCHO<br>10% RH	6.30	This work
CC-3	urea-formaldehyde (UF) resin	25 ppm HCHO<br>dry conditions	4.30	[43]
CAC-800	chitin	10 ppm HCHO<br>dry conditions	4.23	[44]
PMC-A2	pig manure	~2 ppm HCHO<br>dry conditions	0.78	[24]
Commercial activated carbon	coal	2.2 ppm HCHO<br>30% RH	0.08	[16]
Modified commercial activated carbon	coal	2.2 ppm HCHO<br>30% RH	3.80	[16]
CC-D	carbon textile	~1 ppm HCHO<br>dry conditions	1.56	[5]
BAX-M	wood	~1 ppm HCHO<br>dry conditions	0.66	[17]
PSC	poly (sodium 4-styrene sulfonate)	~1 ppm HCHO<br>dry conditions	0.31	[17]
S208	coconut shell	~1 ppm HCHO<br>dry conditions	0.31	[17]

Table S2. The Yoon-Nelson model fitting parameters of pristine BC and NBCs for HCHO adsorption.

Sample	T_{50} (min)	k (min^{-1})	R^2
BC	32.762	0.100	0.935
NBC-4	131.563	0.026	0.996
NBC-8	184.347	0.014	0.980
NBC-12	111.965	0.020	0.980

Table S3. The total yield of pristine BC and NBCs.

Sample	Weigh of jujube pits (g)	Weigh of biochars (g)	yields (%)
BC	3	0.8922 g	29.74
NBC-4	3	1.4666 g	48.89
NBC-8	3	1.6011 g	53.37
NBC-12	3	1.6670 g	55.57

References

[5] Falco, G.; Barczak, M.; Montagnaro, F.; Bandosz, T. A new generation of surface active carbon textiles as reactive adsorbents of indoor formaldehyde. *ACS Appl. Mater. Interfaces* **2018**, *10*, 8066–8076.

[16] Ma, C.; Li, X.; Zhu, T. Removal of low-concentration formaldehyde in air by adsorption on activated carbon modified by hexamethylene diamine. *Carbon* **2011**, *49*, 2873–2875.

[17] Falco, G.; Li, W.; Cimino, S.; Bandosz, T. Role of sulfur and nitrogen surface groups in adsorption of formaldehyde on nanoporous carbons. *Carbon* **2018**, *138*, 283–291.

[24] Suresh, S.; Kante, K.; Fini, E.; Bandosz, T. Combination of alkalinity and porosity enhances formaldehyde adsorption on pig manure-derived composite adsorbents. *Micropor. Mesopor. Mater.* **2019**, *286*, 155–162.

[38] Zhu, X.; Zhang, L.; Zhang, M.; Ma, C. Effect of N-doping on NO_2 adsorption and reduction over activated carbon: An experimental and computational study. *Fuel* **2019**, *258*, 116109.

[39] Li X.; Zhang R.; Zhu X.; Zhang L. Effect of N-doping on the catalytic decomposition of hydrogen iodide over activated carbon: Experimental and DFT studies, *Int. J. Hydrogen Energy* **2020**, *45*, 4511-4520.

[40] Frisch M.; Trucks G.; Schlegel H. et al. Gaussian 16 Revision A. 03, **2016**.

[41] Lu T.; Chen F. Multiwfn: A multifunctional wavefunction analyzer, *J. Comput. Chem.* **2012**, *33*, 580-592.

[42] Humphrey W.; Dalke A.; Schulten K. VMD: visual molecular dynamics, *J. Mol. Graph.* **1996**, *14*, 33-38.

[43] Su C.; Liu K.; Guo J.; Ma W.; Li H; Zeng Z.; Li L. Development of nitrogen-enriched carbon materials by the subtraction method for formaldehyde adsorption, *Surf. Interfaces* **2021**, *24*, 101038.

[44] Ryu D.; Kim D.; Kang Y.; Lee Y.; Nakabayashi K.; Miyawaki J.; Park J.; Yoon S. Preparation of environmental-friendly N-rich chitin-derived activated carbon for the removal of formaldehyde, *Carbon Lett.* **2022**, *32*, 1473-1479.
